# Complete genome sequence of *Tornadovirus japonicus*, a relative of Pacmanvirus, isolated from the Tamagawa River in Japan

**DOI:** 10.1128/mra.00265-24

**Published:** 2024-06-11

**Authors:** Daniel Waschestjuk, Kazuyoshi Murata, Masaharu Takemura

**Affiliations:** 1 Graduate School of Science, Tokyo University of Science, Shinjuku, Tokyo, Japan; 2 Rijksuniversiteit Groningen, Faculty of Science and Engineering, Groningen, the Netherlands; 3 Exploratory Research Center on Life and Living Systems (ExCELLS), National Institute of Natural Sciences, Okazaki, Aichi, Japan; 4 National Institute for Physiological Sciences, National Institutes of Natural Sciences, Okazaki, Aichi, Japan; 5 Department of Physiological Sciences, School of Life Science, Graduate University for Advanced Studies (SOKENDAI), Okazaki, Aichi, Japan; 6 Laboratory of Biology, Institute of Arts and Sciences, Tokyo University of Science, Shinjuku, Tokyo, Japan; Katholieke Universiteit Leuven, Leuven, Belgium

**Keywords:** Nucleocytoviricota, Pacmanvirus, tornadovirus, giant virus, acanthamoeba

## Abstract

Here, we report the isolation and genome sequencing of a new Pacmanvirus-related isolate, *Tornadovirus japonicus*, from the Tamagawa River in Japan. This icosahedral virus has a genome of approximately 380 kb and 465 open reading frames, including two tRNA genes. The name “tornado” is based on its morphological features revealed by transmission electron microscopy analysis.

## ANNOUNCEMENT

Pacmanvirus is a dsDNA virus that infects acanthamoeba. Many Pacmanvirus genes have been reported to be similar to those of faustoviruses and kaumoebaviruses, which are relatives of the family *Asfarviridae* (1). To date, only two strains of pacmanviruses have been isolated from water samples in Algeria (A23 and S19) (1, 2). Here, we report the successful isolation of this virus from water samples collected from the shores of the Tamagawa River in Kawasaki City, Kanagawa Prefecture, Japan (35°58911.77N, 139°65678.59E) on 22 October 2022.

**Fig 1:**
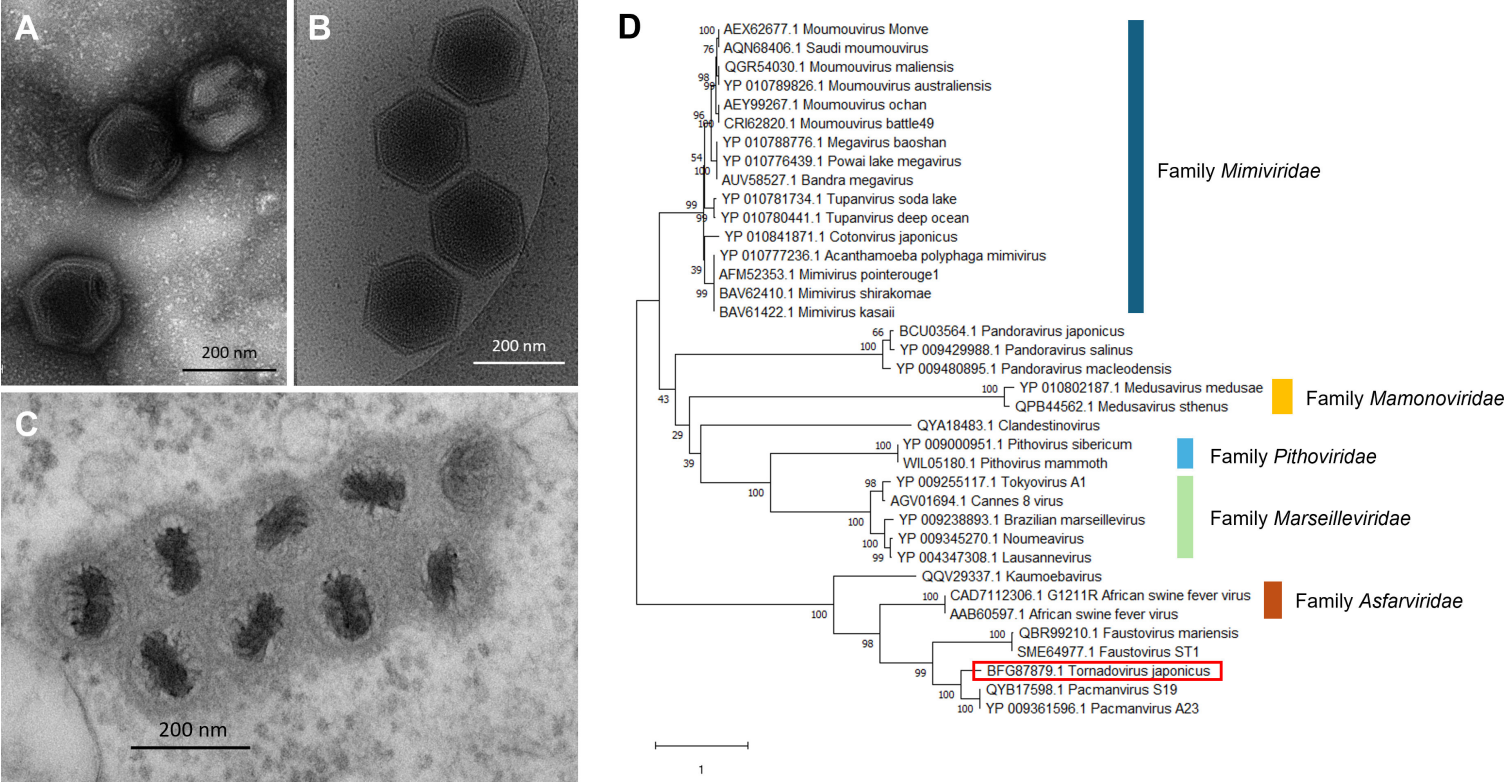
(**A**) Negative-staining electron microscopy (EM) images of *Tornadovirus japonicus* particles. (**B**) Cryo-EM images of *Tornadovirus japonicus* particles. (**C**) TEM image of *Tornadovirus japonicus* particles during maturation of host cells. Bar: 200 nm (A–C). (**D**) Phylogenetic tree of B family DNA polymerase amino acid sequences. Analysis was performed by the MEGA X software (7), using Clustal W algorithm for alignment and the maximum likelihood method with 1,000 replicates for reconstruction of phylogenetic tree. Red box indicates *Tornadovirus japonicus*.

First, water sample was mixed with *A. castllanii* cell suspension in a culture solution of proteose-peptone-yeast extract-glucose (PYG) medium including antibiotics as described previously (3–5). This mixture was added to a 96-well plate and incubated at 26°C. After 12 days, 10 µL of supernatant from a well that displayed cytopathic effects (CPE) of amoeba was inoculated into fresh *A. castellanii* cells and was cultured in 25 cm^2^ culture flasks to propagate the putative virus followed by virus cloning as described previously (6). To identify the viruses, negative-staining electron microscopy was performed using a JEM-2100F operated at 120 kV (JEOL Co. Ltd., Tokyo, Japan) (Fig. 1A). Cryo-electron microscopy was performed using a JEM-2200FS, operated at 200 kV (JEOL Co. Ltd.), which showed the detailed structure of the virus particles (Fig. 1B). To investigate viral formation in *A. castellanii* cells, transmission electron microscopy (TEM) of the virus-infected amoeba cells was performed using a JEM-1400 (JEOL Co. Ltd.) at the Hanaichi UltraStructure Research Institute (Aichi, Japan) (Fig. 1C). Although the specific morphology of the virus remains unknown, we have used here the term “tornado” to describe its unique architecture, based on the morphological features of the maturing viral particles observed in infected cells by TEM (Fig. 1C).

Genomic DNA was extracted from the cloned and propagated tornadoviruses using a NucleoSpin tissue XS (Macherey-Nagel GmbH & Co. KG, Duren, Germany), according to the manufacturer's instructions. A DNA library was prepared for sequencing using a TrueSeq DNA PCR-Free (350) kit (Illumina, Inc.). Sequencing was performed using the NovaSeq 6000 platform (Illumina, Inc.). SPAdes 3.15.0 was used to assemble 28,832,452 reads (4,787,212,192 total read bases) into one contig of 378,863 nucleotides (8). Read length was 151. FastQC (v0.11.5) was used for quality control checks on raw sequence data. Open reading frames (ORFs) were detected using Prokka (9). Gene function was predicted using NCBI BLAST (10). The GC content of the genome was 38.81%. A total of 465 open reading frames were identified, including 463 coding sequences and two tRNAs using NCBI BLAST (10). There were representative genes, which were found in previously reported pacmanviruses, including families B and X DNA polymerases, major capsid proteins, and virion packaging ATPase. Molecular phylogenetic analysis using family B DNA polymerase genes indicated that tornadoviruses are closely related to pacmanviruses with 55.6% identity (Fig. 1D).

## Data Availability

The sequence data are available in GenBank (accession number LC801470.1) under BioProject accession numbers PRJDB17414 and SAMD00735632, and the raw reads can be found in the Sequence Read Archive (accession number DRR527376).
